# Whole-volume ADC Histogram and Texture Analyses of Parotid Glands as an Image Biomarker in Evaluating Disease Activity of Primary Sjögren’s Syndrome

**DOI:** 10.1038/s41598-018-33797-x

**Published:** 2018-10-18

**Authors:** Chen Chu, Fengxian Wang, Huayong Zhang, Yun Zhu, Chun Wang, Weibo Chen, Jian He, Lingyun Sun, Zhengyang Zhou

**Affiliations:** 1 Department of Radiology, Nanjing Drum Tower Hospital, The Affiliated Hospital of Nanjing University Medical School, Nanjing, 210008 China; 2 Department of Rheumatology, Nanjing Drum Tower Hospital, The Affiliated Hospital of Nanjing University Medical School, Nanjing, 210008 China; 3 Philips Healthcare, Shanghai, 200233 China

## Abstract

Diffusion weighted imaging (DWI) has proven to be sensitive for detecting early injury to the parotid gland in pSS (primary Sjögren’s syndrome). Here, we explored the application of ADC histogram and texture analyses for evaluating the disease activity of pSS. A total of 55 patients with pSS who met the classification criteria of the 2002 AECG criteria prospectively underwent 3.0-T magnetic resonance imaging (MRI) including DWI (*b* = 0 and 1000 s/mm^2^). According to the ESSDAI score, 35 patients were categorized into the low-activity group (ESSDAI < 5) and 20 into the moderate-high-activity group (ESSDAI ≥ 5). Via analysis of the whole-volume ADC histogram, the ADC_mean_, skewness, kurtosis, and entropy values of the bilateral parotid glands were determined. Multivariate analysis was used to identify independent risk factors for predicting disease activity. The diagnostic performance of the indexes was evaluated via receiver operating characteristic (ROC) analysis. ROC analysis showed that the anti-SSB, lip biopsy, MRI morphology, ADC, ADC_mean_, and entropy values were able to categorize the disease into two groups, particularly the entropy values. The multivariate model, which included anti-SSB, MRI morphology and entropy, had an area under the ROC curve of 0.923 (P < 0.001). The parotid entropy value distinguished disease activity in patients with pSS, especially combined with anti-SSB and MRI morphology.

## Introduction

Primary Sjögren’s syndrome (pSS) is a chronic autoimmune disease that results in direct injury to the exocrine glands^1,2^. The objective diagnostic evidence of pSS includes serological, ocular, salivary, and labial gland histopathological tests^3^. The salivary tests include salivary flow measurement, X-ray sialography, and technetium-99m pertechnetate scintigraphy. X-ray sialography is a classical examination but is invasive and requires exposure to radiation.

pSS progresses slowly with internal organ damage during the course of the disease. Hence, assessing disease activity is important to for developing an optimized treatment and predicting disease prognosis. Several indexes have been developed to evaluate disease activity and thus reflect the activity of pSS^2,4–6^. The European League Against Rheumatism (EULAR) pSS disease activity index (ESSDAI) score is used as a consensus and includes organ-by-organ definitions, providing a standardized instrument for the homogeneous evaluation of systemic activity in clinical trials and daily practice^2,6,7^. Some recent studies have highlighted the role of imaging modalities for evaluating the disease activity of pSS. Fidelix *et al*. reported that the parotid ultrasonography severity scores were related to the ESSDAI scores in patients with pSS^8^. Cohen *et al*. reported that the 18F-fluorodeoxyglucose positron emission tomography/computed tomography activity score was correlated with ESSDAI and could help to assess disease activity in patients with pSS, resulting in exposure to radionuclides^9^.

Although magnetic resonance imaging (MRI) is not included in the diagnostic criteria of the EULAR for pSS, it has be found to be superior to X-ray sialography for noninvasively evaluating the parotid parenchyma. Conventional, particularly functional MRI provides accurate and abundant information regarding glandular microenvironmental changes, which are characterized by inflammation of the parotid glands. Thus, evaluating parotid injury based on MRI may reflect the disease activity of pSS. Diffusion-weighted imaging (DWI), which quantifies water molecular diffusion with an apparent diffusion coefficient (ADC) value, has been shown to be sensitive for detecting early injury to the parotid glands in pSS^10–12^. Nevertheless, only the mean ADC value has been obtained from one or several regions of interest (ROIs) in most previous studies^10,12^, introducing a sampling error and neglecting the heterogeneity of the parotid injury.

ADC histogram and texture analyses provide a series of parameters, such as skewness, kurtosis and entropy, that can reflect tissue characteristics, such as inflammation, micro-necrosis, oedema and heterogeneity in tissue^13,14^. Whole-volume ADC histogram and texture analyses have been applied in the study of inflammation diseases^15,16^, yet they have not been previously reported for evaluating parotid injury of pSS patients.

Therefore, the purpose of this study was to compare the differences between whole-volume ADC histogram and texture analyses for parotid glands in pSS patients with different activities and to explore the potential of these indexes in predicting disease activity in pSS patients.

## Results

### Differences in clinical and laboratory indexes

The anti-SSB and lip biopsy positive rates in the moderate–high-activity group were significantly higher than those in the low-activity group (P < 0.001 and 0.004, respectively). No significant differences were found in oral dryness, ocular dryness, ANA, anti-SSA, or ocular tests between the two groups (all P > 0.05). There were no significant differences in age and disease duration distribution between the two groups (P = 0.348 and 0.490).

### Difference in parotid MRI morphology

The MRI morphology grades of bilateral parotid glands were consistent for each patient. The moderate–high-activity group had a positive MRI morphology more frequently compared with the low-activity group (80.0% vs. 28.6%, P = 0.001).

### Difference in the parotid ADC value obtained from a ROI

No significant difference was observed in the parotid ADC value obtained from the ROI between the bilateral parotid glands of each patient in any group (P = 0.357 and 0.286). Hence, the average ADC value of the bilateral parotid glands was calculated as the final value for each patient. The parotid ADC value of the moderate-high-activity group was significantly lower than that of the low-activity group (713.6 ± 169.2 vs. 826.5 ± 109.8 × 10^−6^ mm^2^/s, P = 0.004).

### Differences in the parotid whole-volume ADC histogram and texture parameters

No significant differences were found in the whole-volume ADC histogram or texture parameters between the bilateral parotid glands of each patient in any group (P = 0.768, 0.572, 0.521 and 0.478, respectively). Hence, the average ADC histogram parameters of the bilateral glands were calculated as the final values for each patient.

**Figure 1:**
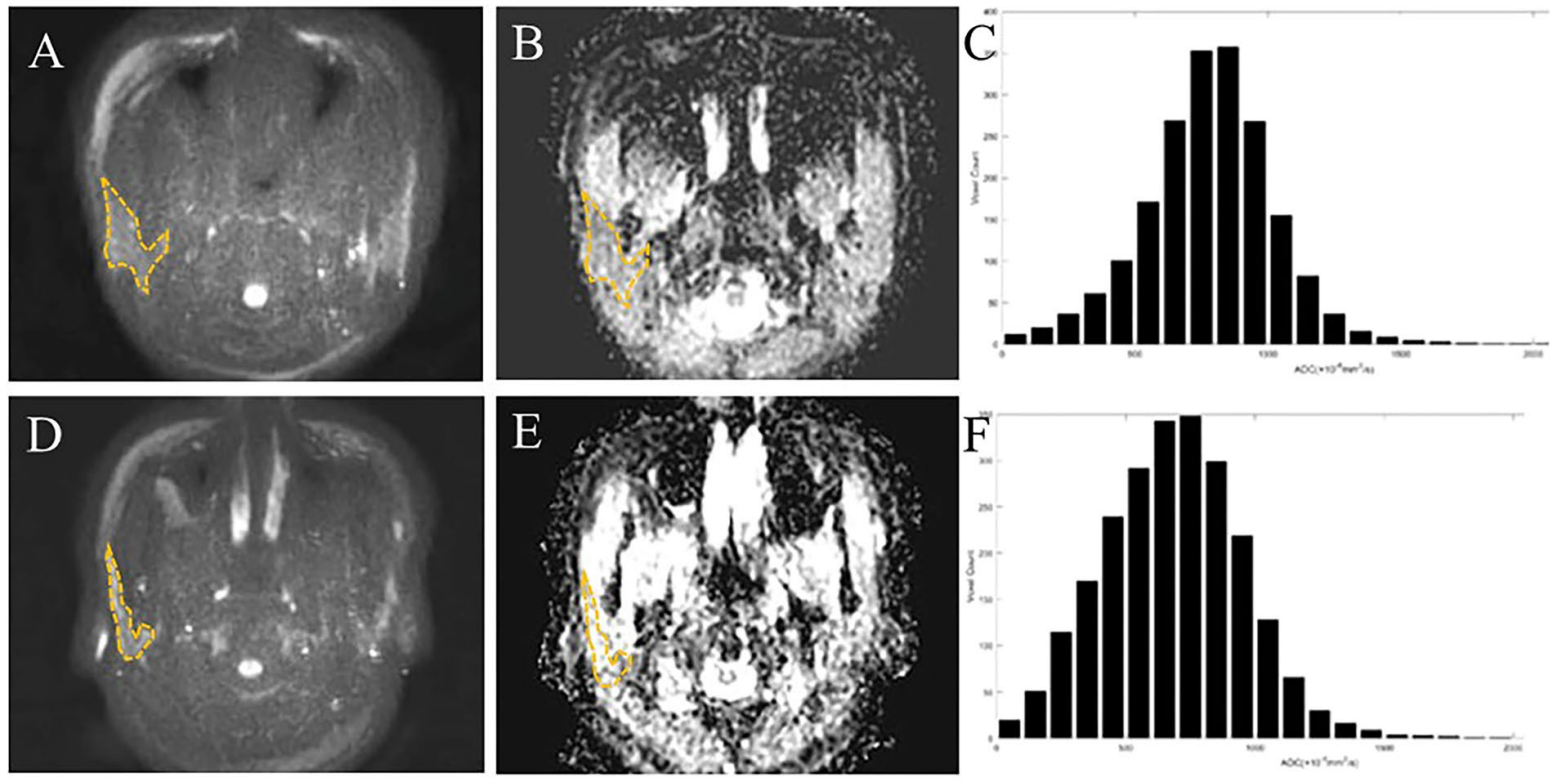
**(A**–**C**) Axial diffusion-weighted imaging (DWI) scan (*b* = 1000 s/mm^2^), corresponding to the apparent diffusion coefficient (ADC) map, and histogram of the bilateral parotid glands of an 18-year-old woman with low-activity primary Sjögren’s syndrome (pSS). Her European League Against Rheumatism (EULAR) pSS disease activity index (ESSDAI) score was 4 (haematological score 2+ serum biomarker score 2). MRI morphology, ANA, anti-SSA, X-ray sialography, and lip biopsy showed positive findings, whereas her anti-SSB and ocular tests were negative. The parotid ADC, ADC_mean_, skewness, kurtosis, and entropy values were 959.1 × 10^−6^ mm^2^/s, 1006.2 × 10^−6^ mm^2^/s, −0.177, 4.746, and 5.690, respectively. (**D**–**F**) Axial DWI scan (*b* = 1000 s/mm^2^), corresponding to the ADC map, and histogram of the bilateral parotid glands in a 66-year-old woman with moderate-activity pSS. Her ESSDAI score was 6 (haematological test, serum biological marker score 2 and constitutional symptoms, articular score 1). MRI morphology, ANA, anti-SSA, anti-SSB, and X-ray sialography showed positive findings, whereas the ocular tests and lip biopsy were negative. The ADC, ADC_mean_, skewness, kurtosis, and entropy values were 943.0 × 10^−6^ mm^2^/s, 1015.6 × 10^−6^ mm^2^/s, 0.359, 5.254, and 6.181, respectively. Note the dashed lines covering the edge of the right parotid glands.

The parotid ADC_mean_ and entropy values of the moderate–high-activity group were significantly lower than those of the low-activity group (P = 0.042, < 0.001, respectively) (Table 1). Representative DWI scans (*b* = 1000 s/mm^2^), corresponding ADC maps, and histograms of two patients are shown in Fig. 1.

**Table 1:** The parotid magnetic resonance imaging (MRI) indexes of patients with primary Sjögren’s syndrome having different activities.

Index	Low-activity group (n = 35)	Moderate–high activity group (n = 20)	P
MRI morphology (positive/negative)	10/25	16/4	0.001^*^
ADC (×10^−6^ mm^2^/s)	826.5 ± 109.8	713.6 ± 169.2	0.004^*^
Parotid volume (mm^3^)	3281.5 ± 1163.5	3904.8 ± 1983.4	0.244
ADC_mean_ (×10^−6^ mm^2^/s)	835.3 ± 125.3	733.0 ± 177.8	0.042^*^
Skewness	0.3 ± 0.4	0.4 ± 0.4	0.484
Kurtosis	4.6 ± 1.2	4.1 ± 1.0	0.094
Entropy	5.9 ± 0.2	6.2 ± 0.3	<0.001^*^

Note, ADC, the mean apparent diffusion coefficient (ADC) value, obtained from one region of interest (ROI) in single slice. ADC_mean_, the mean value of all ADC values within the volume of interest (VOI). Skewness, the histogram asymmetry degree around the mean. Kurtosis, a measurement of the histogram sharpness. Entropy, the distribution of gray levels within the VOI. *P < 0.05.

### Diagnostic performance of various indexes

Univariate analysis showed significant differences for the anti-SSB, lip biopsy, MRI morphology, ADC, ADC_mean_, and entropy values between the moderate-high-activity and low-activity group (all P < 0.05). ROC analysis showed that the anti-SSB, lip biopsy, MRI morphology, ADC, ADC_mean_, and entropy values performed well in differentiating the moderate–high-activity group from the low-activity group. Entropy values had the highest area under the ROC curve (AUC) of 0.853 (Table 2). The multivariate model calibration was assessed with the goodness-of-fit Hosmer-Lemeshow test (P = 0.906). The optimal combination included anti-SSB, MRI morphology and entropy and yielded a sensitivity of 95.0%, specificity of 77.1%, accuracy of 83.6%, and AUC of 0.927 (P < 0.001) (Table 3). In multivariate analysis, the *p* value of lip biopsy, ADC, and ADC_mean_ were 0.069, 0.471 and 0.795, respectively. A McNeil test showed that the AUC of the combined indexes was significantly higher than that of any sole index (anti-SSB, lip biopsy, MRI morphology, ADC, ADC_mean_, and entropy, P = 0.0130,<0.0061, 0.0140, 0.0043, 0.0011, and 0.0268, respectively).

**Table 2:** Diagnostic performance of various indexes in differentiating moderate-high from low activity group in primary Sjögren’s syndrome patients.

Index	Cutoff value	Sensitivity	Specificity	Accuracy	AUC	P
Oral dryness	With oral dryness	100.0%	14.3%	45.5%	0.570	0.382
Ocular dryness	With ocular dryness	45.0%	48.6%	47.3%	0.532	0.694
ANA	≥1:100	80.0%	11.4%	36.4%	0.543	0.600
Anti-SSA	Positive	80.0%	25.7%	45.5%	0.529	0.726
Anti-SSB	Positive	65.0%	88.6%	80.0%	0.768	0.001^*^
X-ray sialography	Rubin and Holt scores positive	90.0%	34.3%	54.5%	0.621	0.137
Ocular tests	Schirmer’s I test ≤ 1.5 mL/15 min or Rose Bengal staining test positive	55.0%	34.3%	41.8%	0.554	0.512
Lip biopsy	Focus score ≥ 1 focus per 4 mm^2^	80.0%	62.9%	69.1%	0.714	0.009^*^
MRI morphology	Grade 1–3	80.0%	71.4%	74.5%	0.757	0.002^*^
ADC	<698.2 × 10^−6^ mm^2^/s	55.0%	88.6%	76.4%	0.696	0.017^*^
Volume	>2806.3 mm^3^	40.0%	77.1%	63.6%	0.579	0.336
ADC_mean_	<826.9 × 10^−6^ mm^2^/s	55.0%	85.7%	74.5%	0.666	0.042^*^
Skewness	>0.463	70.0%	40.0%	50.9%	0.550	0.540
Kurtosis	<5.526	95.0%	31.4%	54.5%	0.620	0.142
Entropy	>6.169	65.0%	94.3%	83.6%	0.853	<0.001^*^

Note, ADC, apparent diffusion coefficient; AUC, area under receiver operating characteristic curve.

^*^P < 0.05. Cutoff values were established by calculating the maximal Youden index (Youden index = sensitivity + specificity − 1).

**Table 3:** The multivariate model for distinguishing moderate-high from low activity group in primary Sjögren’s syndrome patients.

	Log OR	SE	OR	P
Anti-SSB	−1.793	0.893	0.167	0.045
MRI morphology	−1.974	0.921	0.139	0.032
Entropy	5.335	2.132	207.449	0.012

Note, Log OR, Logarithm of odds ratio; OR, odds ratio; SE, standard deviation.

### Correlation between the ADC histogram and texture parameters and the scores for each of the ESSDAI items

As shown in Appendix Table 1, skewness was negatively correlated with cutaneous injury (r = 0.333, P = 0.013) and positively correlated with serum biomarkers (r = 0.307, P = 0.023). Kurtosis was negatively correlated with constitutional symptoms (r = 0.304, P = 0.024) and positively correlated with serum biomarkers (r = 0.340, P = 0.011). Entropy was negatively correlated with muscular injury (r = 0.268, P = 0.018) and positively correlated with constitutional symptoms (r = 0.318, P = 0.048).

### Intra- and interobserver agreements of MRI interpretation

The intra- and interobserver agreements regarding the evaluation of the MRI morphology grade were excellent (kappa coefficient = 0.895 and 0.950, respectively). The measurements of the parotid ADC value and all of the ADC histogram and texture parameters showed excellent intra- and interobserver agreement, with ICCs ranging from 0.899 to 0.982 (Table 4).

**Table 4:** Intra- and interobserver agreement of apparent diffusion coefficient (ADC) value and ADC histogram parameters

Index	Low-activity group		Moderate–high-activity group	
	Intraobserver	Interobserver	Intraobserver	Interobserver
ADC	0.932 (0.902–0.948)	0.956 (0.913–0.974)	0.899 (0.892–0.932)	0.907 (0.893–0.923)
ADC_mean_	0.982 (0.975–0.990)	0.989 (0.974–0.993)	0.908 (0.899–0.923)	0.905 (0.900–0.913)
Skewness	0.915 (0.910–0.918)	0.917 (0.910–0.922)	0.899 (0.889–0.912)	0.932 (0.892–0.951)
Kurtosis	0.924 (0.910–0.953)	0.938 (0.921–0.979)	0.911 (0.899–0.921)	0.915 (0.903–0.922)
Entropy	0.957 (0.950–0.965)	0.935 (0.923–0.942)	0.926 (0.912–0.935)	0.937 (0.921–0.943)

All data in the tables are ICC (95% CI).

## Discussion

This study compared the differences among clinical, laboratory, and imaging indexes between patients with pSS with different levels of disease activity and confirmed the feasibility of using parotid ADC histogram and texture analyses to predict the disease activity of pSS.

The ESSDAI score was 3.4 ± 1.9 in the present study cohort, which was slightly higher than 3.14 ± 3.47 in the report by Fidelix *et al*.^8^. This difference might be because patients with pSS with low disease activity accounted for 63.3% of all patients in the present study, but 72.9% of all patients based on ultrasonography in the study by Fidelix *et al*.^8^.

This study found that the moderate-high-activity group had positive anti-SSB more frequently compared with the low activity-group. Maslinska *et al*.^17^ reported that the presence of anti-SSB was significantly affected by the higher activity of the disease, which was consistent with the findings of the present study. Additionally, the moderate-high-activity group had positive MRI morphology, X-ray sialography, and lip biopsy findings more frequently compared with the low-activity group, which has not been previously reported. We speculate that the parotid and lip glands are more prone to injury in patients with pSS with high disease activity.

The parotid ADC values from one ROI and ADC_mean_ based on the whole-volume histogram and texture analyses were significantly lower in the moderate–high-activity group than in the low-activity group. Previous studies have reported that the parotid ADC value increased during the early stages of pSS due to oedema and the increased vascular permeability of parotid glands^3,18^, but it decreased during the late stages of the disease due to fatty deposition and atrophy of the parotid glands^19,20^. The decreased parotid ADC value in the moderate-high-activity group may also be involved in the decreased microvascular perfusion of the glands. The present study failed to detect a significant difference in the parotid volume between different levels of disease activities.

We found that skewness and kurtosis correlated with some of the ESSDAI items. A lower skewness indicates a higher frequency of high ADC values, which may be due to inflammation and micro-necrosis. A lower kurtosis indicates high heterogeneity of the tissue, which may be due to cell proliferation and necrosis. The entropy value of the parotid glands was significantly higher in the moderate–high-activity group than in the low-activity group. It was speculated that the entropy value might be related to the inflammation characteristics of parotid injury. Texture analysis reflects inflammation disease activity based on the histological characteristics, including transmural inflammation, fissuring ulcers, and oedema^15,21^. Makanyanga *et al*.^15^ found that entropy reflected Crohn’s disease activity according to the microenvironment heterogeneity and complexity. Chu *et al*.^22^ reported that the parotid gland microenvironmental complexity increased with aggravation of the injury grade.

Parotid entropy could distinguish pSS with moderate–high disease activity from pSS with low disease activity with an AUC of 0.853, which was higher than any other single index, including clinical, laboratory, and other imaging parameters. The multivariate model that included parotid entropy, anti-SSB, and MRI morphology yielded a higher value on the goodness-of-fit Hosmer-Lemeshow test. The AUC of this model reached 0.927, with a high sensitivity of 95.0% and relatively low specificity of 77.1%. The disease activity of pSS could be reflected more comprehensively and accurately by combining the information on the parotid glands, serum biomarkers, and lip biopsy.

The fat content and heterogeneity of the parotid glands increase with age, which might affect the DWI-derived parameters. In our study, there was no significant difference in age between the moderate-high-activity and low-activity groups.

Nevertheless, via imaging, the injury has been observed to be significantly advanced in patients with low disease activity and a well-established long-standing disease. Therefore, it is critical to determine the duration of the disease because it is an important confounding factor. Hence, we reviewed the disease duration of all pSS patients. Nevertheless, the disease duration showed no significant differences between the low and moderate-high activity groups. Fidelix *et al*.^8^ also reported no significant correlation between disease duration and ESSDAI, which is consistent with our findings.

### Limitations

The present study had several limitations. First, the sample size was relatively small, although it was larger than previous MRI studies on pSS^10,12,23^. Second, other functional MRI analyses, such as dynamic contrast-enhanced imaging, were not performed or compared with DWI. Third, the diagnostic criteria proposed in our study should be validated in another cohort. Fourth, parotid biopsy was not performed due to its invasiveness and patient discomfort. These issues require further investigation.

## Conclusion

In conclusion, the entropy value derived from whole-volume ADC histogram and texture analyses of the parotid glands shows great potential for predicting the disease activity of pSS. The diagnostic performance of whole-volume ADC histogram and texture analyses can be further improved by combining parotid entropy with anti-SSB and the MRI morphology, which can serve as an imaging biomarker of pSS disease activity.

## Materials and Methods

### Patients

This study was approved by the ethics committee of Nanjing Drum Tower Hospital. Written informed consent was obtained from all subjects. All experiments were performed in accordance with the relevant guidelines and regulations. From October 2016 to August 2017, patients presenting with xerostomia or xerophthalmia were consecutively and prospectively enrolled according to the following criteria: (1) willing to undergo four objective examinations, including serological tests (anti-SSA and anti-SSB with enzyme-linked immunosorbent assay), ocular tests (Schirmer’s I test, positive ≤ 1.5 mL/15 min, and Rose Bengal staining test), labial gland biopsy from the lower lip (positive when the focus score was ≥ 1 focus per 4 mm^2^, and one focus ≥ 50 mononuclear cell aggregation), and X-ray sialography (according to Rubin and Holt scores) to confirm the diagnosis; (2) meeting the 2002 American-European Consensus Group’s (AECG) pSS classification criteria^24^; and (3) no history of the use of glucocorticoid or immune-suppressive agents within the 3 months prior to MRI.

The exclusion criteria were as follows: (1) a history of radiotherapy applied to the head and neck, hepatitis C virus infection, acquired immunodeficiency disease, lymphoma, sarcoidosis, or drug use, such as diuretics, tricyclic antidepressants, and/or anticholinergic agents causing xerostomia (n = 1); (2) a diagnosis of secondary SS associated with other autoimmune diseases, such as systemic lupus erythematosus and rheumatoid arthritis (n = 5); or (3) contraindications to MRI, such as a cardiac pacemaker or artificial cochlear implantation (n = 1).

The two groups significantly differed at the beginning of the study in the glandular domain of the ESSDAI, which was positive in only one patient (2.8%, 1/36) in the low-ESSDAI group and in 31.0% (9/29) rather than 47.7% of patients in the moderate-high ESSDAI group, which was close to the positive rate of 28.12% in Seror R *et al*.’s report^2^ in pSS patients. A Chi-square test showed significant differences in the glandular domain of ESSDAI between the two groups (P = 0.002). ROC analysis showed that the glandular involvement of ESSDAI was able to differentiate the moderate–high-activity group from the low-activity group with a low sensitivity of 31.0%, high specificity of 97.2% and accuracy of 67.7%. To rule out the interference of the glandular domain, we excluded pSS patients with glandular involvement of ESSDAI. Finally, 55 patients were enrolled (53 females and 2 males; mean age, 46.8 ± 14.8 years; age range, 17.0–78.0 years; mean disease duration, 3.8 ± 5.4 years; disease duration range, 0.04 ± 20.0 years), including 50 patients with xerostomia (90.9%), 27 with xerophthalmia (49.1%), 47 with antinuclear antibody (ANA) positivity (85.5%), 42 with anti-SSA positivity (76.4%), 17 with anti-SSB positivity (30.9%), 41 with X-ray sialography positivity (74.5%), 34 with ocular test positivity (61.8%), and 29 with lip biopsy positivity (52.7%).

### pSS disease activity index evaluation

Two rheumatologists (C.W. and H.Y.Z., with 8 and 10 years of experience, respectively) evaluated the pSS disease activity according to the ESSDAI score criteria, including information on constitutional symptoms; lymphadenopathy; glandular, articular, cutaneous, pulmonary, renal, muscular, and peripheral nervous and central nervous system injury; and haematological and serum biomarkers, based on clinical, laboratory, and radiological examinations. Each system was scored as 0 for no activity, 1 for low activity, 2 for middle activity, and 3 for high activity^2^. A third rheumatologist (L.Y.S., with 20 years of experience) was consulted in the case of any inconsistency between the two primary rheumatologists. The ESSDAI score was the sum of 12 systems, which was defined as low (ESSDAI < 5), moderate (5 ≤ ESSDAI ≤ 13), or high (ESSDAI ≥ 14) activity levels^6^. In the present study, patients with low activity served as group 1 and those with moderate or high activity served as group 2. The mean ESSDAI score of the 55 patients was 3.4 ± 1.9 (0–7), including 35 with low activity and 20 with moderate-high activity. The clinical, systemic, and laboratory characteristics of the two groups are shown in Table 5.

**Table 5:** The clinical, systemic, and laboratory characteristics of patients with primary Sjögren’s syndrome having different activities

Index	Low activity (n = 35)	Moderate–high activity (n = 20)
ESSDAI score	2.4 ± 1.4	5.2 ± 1.2
Age (year)	46.6 ± 13.4	47.3 ± 17.5
Disease duration (year)	3.8 ± 5.1	3.8 ± 6.1
Female/male	34/1	19/1
Oral dryness (+)	30 (85.7%)	20 (100.0%)
Ocular dryness (+)	18 (51.4%)	9 (45.0%)
ANA (+)	31 (88.6%)	16 (80.0%)
Anti-SSA (+)	26 (74.3%)	16 (80.0%)
Anti-SSB (+)	4 (11.4%)	13 (65.0%)
X-ray sialography (+)	23 (65.7%)	18 (90.0%)
Ocular tests (+)	23 (65.7%)	11 (55.0%)
Lip biopsy (+)	13 (37.1%)	16 (80.0%)
Constitutional symptoms (+)	4 (11.4%)	12 (60.0%)
Lymphadenopathy (+)	5 (14.3%)	6 (30.0%)
Glandular injury (+)	—	—
Articular injury (+)	4 (11.4%)	2 (10.0%)
Cutaneous injury (+)	3 (8.5%)	3 (15.0%)
Pulmonary injury (+)	4 (11.4%)	9 (45.0%)
Renal injury (+)	2 (5.7%)	0 (0.0%)
Muscular injury (+)	4 (11.4%)	2 (10.0%)
Peripheral nervous system injury (+)	1 (2.9%)	1 (5.0%)
Central nervous system injury (+)	0(0.0%)	0 (0.0%)
Hematological test (+)	13 (37.1%)	15 (75.0%)
Serum biomarkers (+)	20 (57.1%)	16 (80.0%)

Note, ESSDAI, the European League Against Rheumatism (EULAR) SS disease activity index. Constitutional symptoms include fewer, night sweats, and weight loss. Hematological tests include neutropenia, anemia, and thrombocytopenia. Serum biomarkers include C3, C4, CH50, and IgG.

### MRI examination

All patients were asked to fast for 2 h before the MRI examination. Patients were scanned in a supine position head first on a 3.0-T scanner (Ingenia, Philips Medical Systems, Best, The Netherlands) with a 20-channel head and neck joint coil. The MRI scan ranged from the skull base to the submandibular glands, covering the whole volume of the bilateral parotid glands. Participants were asked to minimize their swallowing actions during scanning. The maximum gradient strength and slew rate of the MRI scanner were 45 mT/s and 200 mT/m/s, respectively. The MRI sequences the included axial T1-weighted (T1W) turbo spin-echo [repetition time (TR) = 400–675 ms; echo time (TE) = 18 ms; in-plane resolution = 0.65 × 0.75 mm^2^; field of view (FOV) = 240 × 240 mm^2^; slice thickness = 4.25 mm; intersection gap = 0.67 mm; number of signal averages (NSA) = 2; bandwidth = 287.9 Hz/pixel], axial T2-weighted (T2W) turbo spin-echo (TR = 2500–3500 ms; TE = 90 ms; in-plane resolution = 0.45 × 0.50 mm^2^; FOV = 240 × 240 mm^2^; slice thickness = 4.25 mm; intersection gap = 0.67 mm; NSA = 2; and bandwidth = 217.6 Hz/pixel), axial T2-weighted short tau inversion recovery (STIR) (TR = 3000 ms; TE = 80 ms; in-plane resolution = 0.60 × 0.73 mm^2^; FOV = 240 × 240 mm^2^; slice thickness = 4.25 mm; intersection gap = 0.67 mm; inversion time = 200 ms; NSA = 2; and bandwidth = 228.1 Hz/pixel), and coronal T2-weighted STIR sequences (TR = 1500–2500 ms; TE = 60 ms; acquisition matrix = 2.35 × 3.25 × 4.25; FOV = 200 × 316 mm^2^; slice thickness = 4.25 mm; intersection gap = 0.67 mm; NSA = 1; and bandwidth = 437.1 Hz/pixel). DWI was obtained with a single-shot turbo spin-echo sequence (TR = 5711 ms; TE = 71 ms; number of slices = 15; slice thickness = 4 mm; slice gap = 0.93 mm; FOV = 240 × 240 mm^2^; in-plane resolution = 1.8 × 2.0 mm^2^; acquisition matrix = 132 × 120; reconstruction matrix = 256 × 256; NSA = 4; bandwidth = 819.9 Hz/pixel). Volume shim and high order shim were applied to counter an inhomogeneous B0 field. A DWI with a short TI inversion recovery (STIR) fat suppression was applied and combined with slice-selective gradient reversal (SSGR) to further improve fat suppression^25^. The *b* value used in our article was as reported previously (*b* value = 0, 1000 s/mm^2^)^10,12^. Three motion-probing gradients along the readout, phase-encoding, and slice-selection directions were used. The diffusion registration adopted a 3D affine registration using the “local correlation (LC)” algorithm. The LC algorithm gave a similar or better performance compared with the mutual information algorithm^26^, which avoided various types of subjective movements as well as eddy current effects. The acquisition time of DWI was approximately 3 min 48 s, and the total scan time was approximately 17 min 47 s. All participants underwent MRI successfully without any side effects or discomfort.

### Image analyses

All MRI scans were transmitted to a workstation (Extended MRI WorkSpace 2.6.3.5, Philips Medical Systems, Best, The Netherlands). Two radiologists (C.C. and J.H., with 2 and 10 years of experience in head and neck radiology, respectively) who were blinded to the clinical and laboratory information performed the interpretation and measurement independently. The injury degree of the unilateral parotid gland was evaluated based on T1W, T2W, and T2-STIR images according to the scale proposed by Makula *et al*.^23^ as follows: grade 0, normal homogeneous gland parenchyma; grade 1, fine reticular or small nodular structure with the diameter of nodules <2 mm; grade 2, medium nodular pattern with the diameter of nodules 2–5 mm; and grade 3, coarsely nodular with the diameter of nodules >5 mm. Grade 0 was considered to be negative and grades 1–3 to be positive on MRI for diagnosing SS. A consensus was achieved by discussion if any divergence existed between the two radiologists. The ADC maps were generated from DWI scans with the software integrated within the workstation using a monoexponential model: S = S0 × exp(–*b* × ADC). The DWI scan (*b* = 1000 s/mm^2^) showing that the largest slice of the parotid glands was selected, and an ROI was manually drawn to cover the unilateral parotid gland as large as possible (mean, 464.1 ± 107.5 mm^2^; range, 226.2–723.5 mm^2^), keeping a distance of 1 mm from the boundary and carefully avoiding the retromandibular vein and external carotid artery within the gland. The ROIs were copied to the ADC maps automatically, and the mean ADC value of the ROI was obtained. The mean value of the bilateral parotid glands was calculated.

Whole-volume ADC histogram and texture analyses were performed using in-house software (Image Analyzer 2.0, China), the details of which were described in previous studies^13,27–29^. A series of ROIs were manually drawn to cover the parotid gland as large as possible on each slice of the DWI scan (*b* = 1000 s/mm^2^). The ROIs were copied to the ADC maps automatically. After selecting all of the ROIs of the unilateral parotid gland (slice number, 5–10; mean, 8 ± 1), the volume of interest (VOI) was composed (volume range, 1060.2–8927.8 mm^3^; mean, 3710.6 ± 1442.2 mm^3^) to obtain the following parameters, which were calculated using the following formulas (1–4), where *X* indicates the set of all ADC values, *N* is the number of sampled ADC pixels, $\bar{X}$ is the mean of *X*, and *P*(*i*) is the frequency of voxels with intensity *i* divided by *N*.

1. ADC_mean_, the mean value of all ADC values within the VOI, $$\frac{1}{N}\sum \begin{array}{c}N\\ i\end{array}X(i)$$;
2. skewness, the histogram asymmetry degree around the mean, $\frac{\frac{1}{N}{\sum }_{i=1}^{N}{(X(i)-\bar{X})}^{3}}{{((\sqrt{\frac{1}{N}{\sum }_{i=1}^{N}{({\rm{X}}({\rm{i}})-\bar{X})}^{2}}))}^{3}}$;
3. kurtosis, a measurement of the histogram sharpness, $\frac{\frac{1}{N}{\sum }_{i=1}^{N}{(X(i)-\bar{X})}^{4}}{{((\sqrt{\frac{1}{N}}{\sum }_{i=1}^{N}(X(i)-\bar{X})))}^{2}}$;
4. entropy, the distribution of grey levels over the VOI, $$-\sum \begin{array}{c}{N}_{l}\\ i=1\end{array}P(i){\mathrm{log}}_{2}P(i)$$.

The measurements of each radiologist were recorded separately for interobserver agreement analysis. The mean value of the two radiologists was calculated as the final value of each patient. One radiologist (X.X) repeated all of the measurements 1 month later for intraobserver agreement analysis.

### Statistical analyses

Quantitative data showing a normal distribution (Kolmogorov-Smirnov test, all P > 0.05) are presented as the mean ± standard deviation, and qualitative data are presented as ratios. Continuous variables were compared with two independent-samples t tests, and categorical variables were compared with the Chi-square test between the two groups. The Mann-Whitney U test was used to compare the differences in age and disease duration distribution. The diagnostic performance of each index was tested via receiver operating characteristic (ROC) analysis. Cutoff values were established by calculating the maximal Youden index (Youden index = sensitivity + specificity − 1). Moderate-high activity was defined as a positive result. Univariate analysis was used to compare the indexes for differentiating moderate-high-activity from the low-activity group. Binary logistic regression analysis with a backward stepwise selection procedure was performed to identify the independent predictors for differentiating the moderate-high from the low-activity group. Multivariate model calibration was assessed with the goodness-of-fit Hosmer-Lemeshow test and graphical decile group probability through a calibration plot. The McNeil test was used to compare the area under ROC curves (AUCs). The Spearman rank test was used to correlate the ADC histogram parameters and the scores for each of the ESSDAI items. The kappa coefficient was calculated to evaluate the intra- and interobserver agreements in MRI morphology assessment. Intra- and interobserver agreements in the measurement of ADC parameters were estimated by calculating the intraclass correlation coefficients (ICCs) (0.000–0.200, poor; 0.201–0.400, fair; 0.301–0.600, moderate; 0.601–0.800, good; 0.801–1.000, excellent). Statistical analyses were performed using SPSS (version 22.0 for Microsoft Windows x64, SPSS, IL, USA). A two-tailed P value less than 0.05 was considered statistically significant, and a P value less than 0.05 was considered significant in univariate analysis.

## Electronic supplementary material

Appendix Table 1

## Data Availability

The datasets generated during and/or analysed during the current study are available from the corresponding author on reasonable request.
