# Supplementary material for: Whole-volume ADC Histogram and Texture Analyses of Parotid Glands as an Image Biomarker in Evaluating Disease Activity of Primary Sjögren’s Syndrome
Source: Sci Rep. 2018 Oct 18;8:15387. doi: 10.1038/s41598-018-33797-x (PMC6193973; doi:10.1038/s41598-018-33797-x)
Supplement: Supplementary file 1 — Appendix Table 1 [file 41598_2018_33797_MOESM1_ESM.docx]

Whole-volume ADC Histogram and Texture Analyses of Parotid Glands as an Image Biomarker in Evaluating Disease Activity of Primary Sjögren's Syndrome

Chen Chu, MD, PhD^1, †^, Fengxian Wang, MD^1, †^, Huayong Zhang, MD, PhD^2^, Yun Zhu, PhD^2^, Chun Wang, PhD^2^, Weibo Chen, PhD^3^, Jian He, MD, PhD^1, *^, Lingyun Sun, MD, PhD^2, *^, Zhengyang Zhou, MD, PhD^1, *^

^1^ Department of Radiology, Nanjing Drum Tower Hospital, The Affiliated Hospital of Nanjing University Medical School, Nanjing, China, 210008

^2^ Department of Rheumatology, Nanjing Drum Tower Hospital, The Affiliated Hospital of Nanjing University Medical School, Nanjing, China, 210008

^3^ Philips Healthcare, Shanghai, China, 200233

^†^ Both authors contributed equally to this manuscript.

^*^ Corresponding authors: Jian He (hjxueren@126.com), Lingyun Sun ([lysun_nju@163.com](mailto:lysun_nju@163.com)), and Zhengyang Zhou (zyzhou@nju.edu.cn).

**Appendix Table 1**

Correlation between apparent diffusion coefficient (ADC) histogram parameters and the scores for each of the European League Against Rheumatism primary Sjögren’s syndrome disease activity index (ESSDAI) items using spearman rank test

| Parameter | ADC_mean_ | Skewness | Kurtosis | Entropy |
| --- | --- | --- | --- | --- |
| Constitutional symptoms | -0.168 | -0.039 | -0.304* | 0.318* |
| Lymphadenopathy | -0.248 | 0.253 | 0.030 | 0.263 |
| Glandular injury | ― | ― | ― | ― |
| Articular injury | 0.040 | -0.084 | 0.077 | 0.092 |
| Cutaneous injury | -0.010 | -0.333* | -0.118 | 0.069 |
| Pulmonary injury | -0.118 | 0.208 | 0.073 | 0.223 |
| Renal injury | 0.177 | -0.067 | 0.086 | -0.147 |
| Muscular injury | 0.062 | -0.062 | 0.012 | -0.268* |
| Peripheral nervous system injury | 0.055 | 0.018 | -0.086 | -0.159 |
| Central nervous system injury | ― | ― | ― | ― |
| Hematological test | -0.058 | -0.029 | 0.050 | 0.129 |
| Serum biomarkers | 0.090 | 0.307* | 0.340* | 0.215 |

*, *p* < 0.05; **, *p* < 0.005.
